# Population-based *Neisseria gonorrhoeae*, *Chlamydia trachomatis* and *Trichomonas vaginalis* prevalence using discarded, deidentified urine specimens previously collected for drug testing

**DOI:** 10.1136/sextrans-2017-053355

**Published:** 2017-10-24

**Authors:** Judith Harbertson, Matthew Jamerson, Paul C F Graf, Lisa Kennemur, Brent House, Nelson L Michael, Paul Scott, Brad Hale

**Affiliations:** 1 US Military HIV Research Program, Walter Reed Army Institute of Research, San Diego, California, USA; 2 Health Sciences Business Unit, Leidos, Inc, San Diego, California, USA; 3 Department of Defense HIV/AIDS Prevention Program (DHAPP), Naval Health Research Center, San Diego, California, USA; 4 Navy Drug Screening Laboratory, US Navy, San Diego, California, USA; 5 Naval Medical Center, San Diego, California, USA; 6 Operational Infectious Diseases Department, Naval Health Research Center, San Diego, California, USA; 7 University of California, San Diego, California, USA

**Keywords:** chlamydia trachomatis, neisseriagonorrhoeae

We used a novel method to test for STIs among a non–healthcare-seeking military population in the San Diego region of California. Active-duty US Navy and Marine Corps personnel were randomly selected to provide urine specimens to Navy Drug Screening Laboratory, San Diego in October and November 2013 for the Department of Defense drug testing programme. If specimens screened negative for drugs (>99% of samples), urine specimens were discarded, deidentified and subsequently tested for *Chlamydia trachomatis* (CT), *Neisseria gonorrhoeae* (GC) and *Trichomonas vaginalis* (TV) using the Aptima Combo 2 and TV assay as specified by the manufacturer (Hologic, San Diego, CA, USA). The Tigris direct tube sampling system was used for high-throughput nucleic acid amplification testing (NAAT). Urine specimens older than 6 days were not tested due to sample degradation concerns.

The overall prevalence of CT was 2.1% (95% CI 1.79 to 2.36, n=205/9953), GC 0.01% (95% CI 0.00 to 0.07, n=1/9953) and TV 0.12% (95% CI 0.03 to 0.38, n=3/2553).

Prevalence was not significantly different from civilian estimates.^1^ Prior military data show higher prevalence^2^ but include higher prevalence groups (recruits, larger proportion of female or African American race/ethnicity) not comparable to the regional military population. In the USA, TV prevalence is higher in women than men (3.2% vs 1.9%, see online supplementary material), African Americans than Whites (6.9% vs 1.2%) and those ≥25 than 18–20 year olds (4% vs 1.5%) which may partially account for the low TV prevalence (0.12%); the military population is predominantly male (86%), white (69%) and young (51% ≤25 years old).

10.1136/sextrans-2017-053355.supp1 Supplementary file 1

This testing method was effective for obtaining a standardised STI prevalence in a non–healthcare-seeking US military population using specimens previously collected for drug testing. This method could be adjusted for monitoring changes in STI prevalence, or for measuring asymptomatic infections within the active-duty military population.

## References

[1] Torrone E, Papp J, Weinstock H Centers for Disease Control and Prevention (CDC). Prevalence of *Chlamydia trachomatis* genital infection among persons aged 14-39 years-United States, 2007-2012. MMWR Morb Mortal Wkly Rep 2014;63:834–7. 25254560 PMC4584673

[2] Rietmeijer CA, Hopkins E, Geisler WM, et al Chlamydia trachomatis positivity rates among men tested in selected venues in the United States: a review of the recent literature. Sex Transm Dis 2008;35(11 Suppl):S8–S18. doi:10.1097/OLQ.0b013e31816938ba 18449072 10.1097/OLQ.0b013e31816938ba

