# Supplementary material for: Population-based Neisseria gonorrhoeae, Chlamydia trachomatis and Trichomonas vaginalis prevalence using discarded, deidentified urine specimens previously collected for drug testing
Source: Sex Transm Infect. 2017 Oct 24;94(2):123. doi: 10.1136/sextrans-2017-053355 (PMC5870458; doi:10.1136/sextrans-2017-053355)
Supplement: Supplementary file 1 [file sextrans-2017-053355supp001.docx]

**Supplementary Material**

**REFERENCES**

1. Catania M, Bennett C, Pae A, et al. Clinical titers and stability of *Trichomonas vaginalis* RNA in urine specimens. Poster presented at: International Society for Sexually Transmitted Diseases Research Conference; July 2011; Québec City, Canada.
2. Satterwhite CL, Torrone E, Meites E, et al. Sexually transmitted infections among US women and men: prevalence and incidence estimates, 2008. *Sex Transm Dis* 2013;40:187-193.
3. Miller WC, Swygard H, Hobbs MM, et al. The prevalence of trichomoniasis in young adults in the United States. *Sex Transm Dis* 2005;32:593-8.
4. U.S. Department of Defense. 2013 demographics profile of the military community. 2015. Available at: http://www.militaryonesource.mil/12038/MOS/Reports/2013-Demographics-Report.pdf. Accessed June 1, 2016.
